# Complete genome sequences of AZ *Arthrobacter* phages Wildwest and Sue2

**DOI:** 10.1128/mra.01078-24

**Published:** 2025-02-03

**Authors:** Isabella E. Cloud, Ava N. Ortega, Angelina M. Spencer, Varsha Upadhyayulla, Tamarah L. Adair

**Affiliations:** 1 Department of Biology, Baylor University, Waco, Texas, USA; Queens College Department of Biology, Queens, New York, USA

**Keywords:** bacteriophages, *Arthrobacter*, soil microbiology, science education, Arthrobacterphage

## Abstract

This announcement reports the complete genome sequences of two bacteriophages isolated from soil samples using the host *Arthrobacter atrocyaneus* Strain NRRL B-2883. These findings enhance our understanding of AZ1 cluster phages, particularly Wildwest and Sue2, with their unique genomic features.

## ANNOUNCEMENT

Bacteriophage genome annotation contributes to understanding the role of phages in their environment, the discovery of genes with unknown functions, and aspects of host-viral interactions. *Arthrobacter* is a common environmental organism. It is a member of the Family *Micrococcaceae* and Class *Actinobacteria*, known to survive in numerous stressful environments (1–3). *Arthrobacter atrocyaneus* Strain NRRL B-2883 was used in this study. As of November 2024, only eight phages isolated on *A. atrocyaneus* have been sequenced (4), all assigned to the AZ1 subcluster. This genome report enhances the understanding of AZ phages (5, 6).

BLASTn identifies the top match for Wildwest as *Manhattanvirus drsierra* (7) and for Sue2, *Manhattanvirus kealli* (8), placing both in the Genus *Manhattanvirus*, as described by the International Committee on the Taxonomy of Viruses (9) (Table 1).

**TABLE 1:** Sample isolation data and genome characteristics for Wildwest and Sue2

Phage descriptions	Wildwest	Sue2
Isolation location (soil sample)	St. Charles, Missouri (38.716786 N, 90.477583 W)	Elmhurst, Illinois (41.881132 N, 87.923636 W)
ICTV taxonomy (9)	Duplodnaviria; Heunggongvirae; Uroviricota; Caudoviricetes; subfamily Azeevirinae, Genus Manhattanvirus	Duplodnaviria; Heunggongvirae; Uroviricota; Caudoviricetes; subfamily Azeevirinae, Genus Manhattanvirus
Genome size (base pairs)	43,653	42,245
% GC	66.9	66.6
Number of reads	566,827	226,930
Coverage	1,849×	772×
Number of predicted genes	65	67
NCBI BLASTn % identity with *Manhattenvirus drsierra* (7)	90.96	80.61
NCBI BLASTn % identity with *Manhattenvirus kealli* (8)	81.52	82.68
NCBI BLASTn % identity between Wildwest and Sue2	81.99	81.99

Wildwest and Sue2 were isolated from washed soil samples (Table 1). Soil extracts were filtered through a 0.22 µm filter and plated with *A. atrocyaneus* using Peptone-Yeast-Calcium (4.5 mM) Dextrose (0.1%) top agar (0.4%) and incubated at 32°C for 24–48 hours, as described (10). Purification utilized three rounds of plaque assays, followed by amplification. Phage lysates were collected using webbed plates with final concentrations over 10^9^ pfu/mL. Images of plaques and Siphoviridae virion size and morphology for Sue2 and Wildwest may be seen on PhagesDB (4).

Genomic DNA was isolated using the Wizard DNA Clean-Up Kit (Promega) and sequenced by the Pittsburgh Bacteriophage Institute on an Illumina MiSeq. The NEB Ultra II Library Kit (v3 Reagents) was used with raw read lengths of 150-base single-end reads. Reads were assembled using Newbler 2.9 (11) and Consed 29 (12). Genomic termini were determined as previously described (13).

Auto-annotation was performed using DNAMaster v5.23.6–5.0.2 (14) and PECAAN v.1.2-v2024030 (15), with Glimmer v3.02 (16) and Genmark v3.40 (17) used to predict coding regions. Phamerator v1.2 (18) and Starterator v1.2 (19) verified probable start sites and enabled comparative analysis. HHPred v2.0.13 (20) and BLASTp v2.14-v2.15 (21) were used in the prediction of putative gene functions. Aragorn v1.2.41 (22) and tRNAscan-Se v2.0 (23) were used to detect tRNA genes. Transmembrane proteins were predicted using DeepTmHmm v2.0 (24) and TOPCONS 2.0 (25). All software was used with default settings.

**Fig 1:**
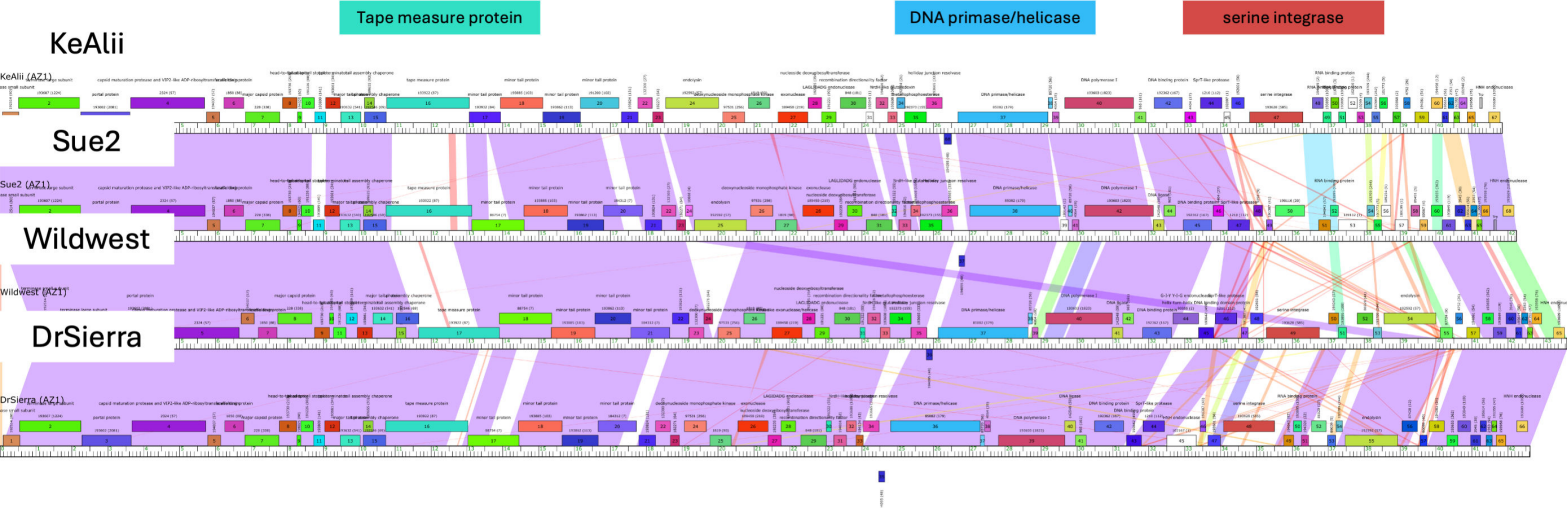
Genome alignment of Sue2 and Wildwest and their closest matches.

Genome characteristics and read counts are in Table 1. Both phages possess a 3’ sticky overhang genome end. Protein coding genes were predicted using BLASTp (21), Phamerator (18), and PhagesDB (4), revealing varying levels of conservation, as seen in the genome alignment (Fig. 1) between Sue2 and Wildwest and their most similar BLASTn matches, KeAlli and DrSierra, respectively. Darker colors between genomes indicate higher nucleotide similarity, and colored boxes mark conserved “phamilies” (phams) based on similarities between protein sequences, illustrating genetic diversity and the mosaic nature of even closely related phages. Three examples are noted. (i) Tape measure protein: conserved pham but variable nucleotide sequence, (ii) DNA primase/helicase: conserved, and (iii) serine integrase: diverse nucleotide sequence, conserved pham, and absent in Sue2. One Trp tRNA was detected within Sue2, but not Wildwest.

## Data Availability

GenBank and Sequence Read Archive accession numbers for Wildwest are OR521060.1 and SRX20630261 and for Sue2 PQ114743 and SRX25029065.

## AFTER EPUB

*[This article was published on 3 February 2025 with an error in Table 1. The Genome size (base pairs) was corrected for Sue2 in the current version, posted on 10 February 2025.]*
